# Investigating blood–brain barrier penetration and neurotoxicity of natural products for central nervous system drug development

**DOI:** 10.1038/s41598-025-90888-2

**Published:** 2025-03-03

**Authors:** Rintaro Kato, Li Zhang, Nivedita Kinatukara, Ruili Huang, Abhinav Asthana, Claire Weber, Menghang Xia, Xin Xu, Pranav Shah

**Affiliations:** National Center for Advancing Translational Sciences (NCATS), 9808 Medical Center Drive, Rockville, MD 20850 USA

**Keywords:** Natural products (NPs), PAMPA-BBB, CNS drug discovery, Neurotoxicity, Neurite outgrowth assay, Drug discovery, Neuroscience, Chemistry

## Abstract

Natural Products (NPs) are increasingly utilized worldwide for their potential therapeutic benefits, including central nervous system (CNS) disorders. Studies have shown açai berries mitigating Parkinson’s disease progression through dopaminergic neuroprotection via Nrf-2 HO-1 pathways. Ashwagandha, an evergreen shrub, has shown potential as a therapeutic for neurodegenerative disorders via axonal regeneration in Aβ25-35-treated cortical neurons in vitro. In most cases, promising NPs are tested using in vitro assays or simpler systems during the early stages of drug discovery. However, a critical challenge lies in the lack of data on blood-brain barrier (BBB) penetration, which is a significant determinant for the successful development of CNS drugs. Our first goal was to test our in-house NP constituent library via the Parallel Artificial Membrane Permeability Assay (PAMPA-BBB), with the aim of understanding their BBB-penetration potential. Of the constituents tested, 255 were found to have moderate to high BBB permeability. Our next goal was to understand if these compounds could exhibit CNS toxicity. Neuronal viability and neurite outgrowth assays were performed with this subset to identify compounds with neurotoxicity potential. Around 35% of compounds tested showed neurite outgrowth inhibition. The habitual and widespread consumption of NPs underscores the importance of subjecting this subset of compounds to additional testing and validation in vivo to ascertain their potential detrimental effects. Understanding BBB permeability and assessing neurotoxicity mechanisms of NPs will significantly benefit the CNS drug discovery community.

**Supplementary Information:**

The online version contains supplementary material available at 10.1038/s41598-025-90888-2.

## Introduction

Historically, natural products (NPs) have played a significant role in drug discovery. Approximately 40% of pharmaceutical products today are drawn from NP knowledge. These include aspirin (headaches), morphine (pain), metformin (diabetes), paclitaxel (cancer), digoxin, and lovastatin (cardiovascular)^1,2^. *Hypericum perforatum L*., commonly known as St. John’s Wort in literature, has been studied for antidepressant^3,4^, and wound healing effects^5^. Hypericin, an NP constituent derived from St. John’s wort, has also exhibited anticancer and neuroprotective properties^6^. NPs have shown multiple facets of therapeutic potential and have been widely investigated to develop anticancer^7^ and antiviral drugs^8^. NPs even play a major role in central nervous system (CNS) diseases with ~ 84% of approved drugs for CNS disease being NPs or NP-inspired^9^. NPs are being investigated for treatments of various ailments including depression^4,6,10^ and neurodegenerative diseases such as: Parkinson’s, Alzheimer’s, Huntington’s, and Amyotrophic lateral sclerosis^11^.

Despite the increasing interest in new pharmacotherapies utilizing NPs, researchers still must overcome the same obstacles during the drug development process, such as metabolic stability, permeability, efficacy, safety, and toxicity^7^. CNS drug development is even more difficult, because the blood brain barrier (BBB) is a highly effective barrier with less than 2% of drugs reaching the CNS^12^. BBB permeability can be assessed in three general ways: in vivo using animal models, in vitro cell-based, and in vitro non-cellular assays. Common cell-based in vitro assays include Madin-Darby Canine Kidney cells (MDCK-MDR1) and human colon adenocarcinoma derived Caco-2, which model active and passive transport, and Organ-on-a-chip assay, which uses microfluidic chips to mimic organ function. The human BBB-Chip, using iPSC-derived brain microvascular endothelial-like cells (iBMECs), replicates brain vasculature and predicts BBB permeability. While these assays are effective, they are labor-intensive, costly, and not easily automated. In contrast, the Parallel Artificial Membrane Permeability Assay (PAMPA) is a simple, cost-effective, high-throughput, and fully automatable assay. Although it cannot assess active efflux transport, this is less of a limitation since most CNS drugs diffuse passively. Our recent findings demonstrate that the PAMPA-BBB assay exhibits a strong correlation with preclinical in vivo brain data. The low cost, high-throughput capability of PAMPA, combined with its flexibility in experimental conditions (such as varying lipid compositions and pH ranges), makes it an excellent screening tool in early drug discovery^13^.

NPs and supplements are generally considered to be safe, however, unlike conventional drugs, they are not regulated for purity and potency^14^. This is seen from 2004 to 2016 when the FDA recorded more than 50,000 adverse events due to NPs and supplements^15,16^. Some of these adverse effects are CNS-related, and symptoms include numbness of limbs, dizziness, headaches twitching, stupor, coma, confusion, respiratory failure, and alopecia^7,17^. According to the World Health Organization, almost half of the population in industrialized countries regularly utilize herbal medicinal products and supplements: 42% in the United States, 49% in France, 70% in Canada^18^, and 75% in South Korea^19^. The habitual use of NPs in combination with conventional drugs, their poor monitoring, and the FDA’s limited post-market enforcement play a part in these adverse reactions and interactions^20^.

The goal of our study is to develop effective approaches for assessing BBB penetration and neurotoxicity potential of diverse and structurally complex NPs to streamline CNS drug discovery and address challenges in their therapeutic application. In this study, we assessed the brain penetration potential of our 1700 NP constituent compound library using the PAMPA-BBB assay and found 255 constituents with moderate to high BBB permeability. Additionally, we evaluated the neurotoxicity potential of this subset of constituents via the neurite outgrowth inhibition assay^21^. Green fluorescent protein (GFP)-labeled induced pluripotent stem cell (iPSC)-derived human cortical glutamatergic neurons were used to detect compound-mediated inhibition of neurite outgrowth. Of the compounds tested, 83 showed potential for neurotoxicity with 53% of constituents showing AC_50_ values < 10 µM. The complete PAMPA-BBB and neurite outgrowth datasets have been made publicly accessible. Furthermore, the PAMPA-BBB model on the NCATS Open Data ADME portal (https://opendata.ncats.nih.gov/adme/) could be re-trained with this new data, enhancing the model’s diversity and ability to serve as a rank-ordering tool in early drug discovery.

## Materials and methods

### Materials

Dimethyl sulfoxide (DMSO, high performance liquid chromatography (HPLC) grade), caffeine, progesterone, rotenone and carbamazepine were purchased from Sigma-Aldrich (St. Louis, MO). Brain sink buffer (Catalog #110674), BBB-1 lipid solution (Catalog #110672), 96-well stirwell sandwich plates (Catalog #110243), and preloaded support plate (for use with 96-well stirwell sandwich plate (Catalog #120551-Supp)) were purchased from Pion Inc. (Billerica, MA). UV plates (Catalog #675801) were purchased from Greiner BIO-ONE (Monroe, NC). 0.5 M potassium phosphate buffer solution, pH 7.4 (Catalog #J61413) was purchased from Thermo Fisher Scientific (Waltham, MA).

Human Cortical Glutamatergic Neurons were bought from (CAG-GFP, Lot# 230714D, BrainXell, Inc). DMEM/F12 Medium, Neurobasal medium, B27 supplement, N2 supplement, GlutaMAX, Geltrex were purchased from Thermo Fisher Scientific, Inc. (Grand Island, NY, USA). BDNF, GDNF and TGF-β1 were purchased from Peprotech (Rocky Hill, NJ, USA). Neuron Supplement was provided by BrainXell, Inc.

### PAMPA-BBB permeability assay

The stirring Double-Sink™ PAMPA-BBB method, patented by Pion Inc. (Billerica, MA), was employed to determine the permeability of compounds^22^. The proprietary PAMPA lipid membrane, which consists of porcine brain lipid extract dissolved in alkane (Pion Inc.), was optimized to predict BBB passive permeability. This membrane was immobilized on a PVDF matrix of a 96-well “acceptor” filter plate placed on top of a 96-well “donor” plate containing coated magnetic stirrers. The test articles, stocked in 10 mM DMSO solutions, were diluted to 0.05 mM in aqueous phosphate buffer and the concentration of DMSO was 0.5% in the final solution. During the 60-minute permeation study period, conducted at room temperature, the test samples in the donor compartment were stirred using the Gutbox technology (Pion Inc.) to reduce the aqueous boundary layer to 60 μm. The test article concentrations in the “donor” and “acceptor” compartments were measured using a UV plate reader (Nano Quant, Infinite^®^ 200 PRO, Tecan Inc., Männedorf, Switzerland). Permeability (P_e_) calculations were performed using Pion Inc. software and were expressed in units of 10^− 6^cm/s.

### Neurite outgrowth inhibition assay in iPSC derived GFP-labeled human neurons

The neurite outgrowth assay was performed in a 1536-well plate format as described previously^23^. Briefly, GFP-expressing human cortical glutamatergic neurons were thawed with freshly made seeding medium according to the recipe provided by BrainXell, Inc., then dispensed at 800 cells/5 µL/well by BioRAPTR into low-base PDL-coated 1536-well black-wall/clear-bottom assay plates (Aurora Microplates, Whitefish, MT). The assay plates were incubated at 37 °C/5% CO_2_ for 42 h, followed by an addition of 23 nL compounds via a Wako Pintool station (Wako Automation, San Diego, CA). The final concentrations of the compounds ranged from 0.4 nM to 92 µM. Rotenone, a known neurite outgrowth inhibitor, was used as a positive control, and DMSO was used as a negative control in the screening. The plates were continuously incubated at 37 °C/5% CO_2_ for 24 to 48 h. Fluorescence intensities (460–490 nm excitation, 500–550 nm emission for EGFP) were measured, and images were acquired through Operetta CLS (Perkin Elmer, Shelton, CT) High-Content Imaging System Harmony 4.6, using 20×water, confocal, and one field for each well. Images were analyzed with the corresponding Operetta CLS software. Several parameters such as total neurite length, root number, and maximum neurite length were used for quantitative image analysis of neurite outgrowth. The number of objects were measured to indicate the number of cells. After imaging the 48 h timepoint, 4 µL CellTitre-glo was added to each well by BioRAPTR, the plates were kept at room temperature for 30 min, and luminescence was measured for cell viability.

### Data analysis

Total neurite length was used to evaluate the inhibitory effects of compounds on neurite outgrowth. The number of objects was used to indicate cell viability. Analysis of compound concentration-response data was performed as previously described^21^. Briefly, raw plate measurements for each titration point were first normalized relative to the positive control compound (46 µM rotenone for cell based assays; 100%) and DMSO-only wells (0%) as follows: Activity (%) = ([V_compound_ – V_DMSO_]/[V_DMSO_ –V_pos_]) × 100, where V_compound_ denotes the compound well values, V_pos_ denotes the median value of the positive control (46 µM rotenone) wells, and V_DMSO_ denotes the median values of the DMSO-only wells. The dataset was then corrected by applying an in-house pattern correction algorithm^24^. The pattern correction algorithm is applied to remove background patterns in the microplates, such as edge effects caused by solvent evaporation and subtle abnormalities such as tip effects or blotting from cell dispenses. The half-maximum activity values (AC_50_) for each compound and maximum response (efficacy) values were obtained by fitting the concentration-response curves of each compound to a four-parameter Hill equation.

## Results

### Dataset distribution

**Fig. 1:**
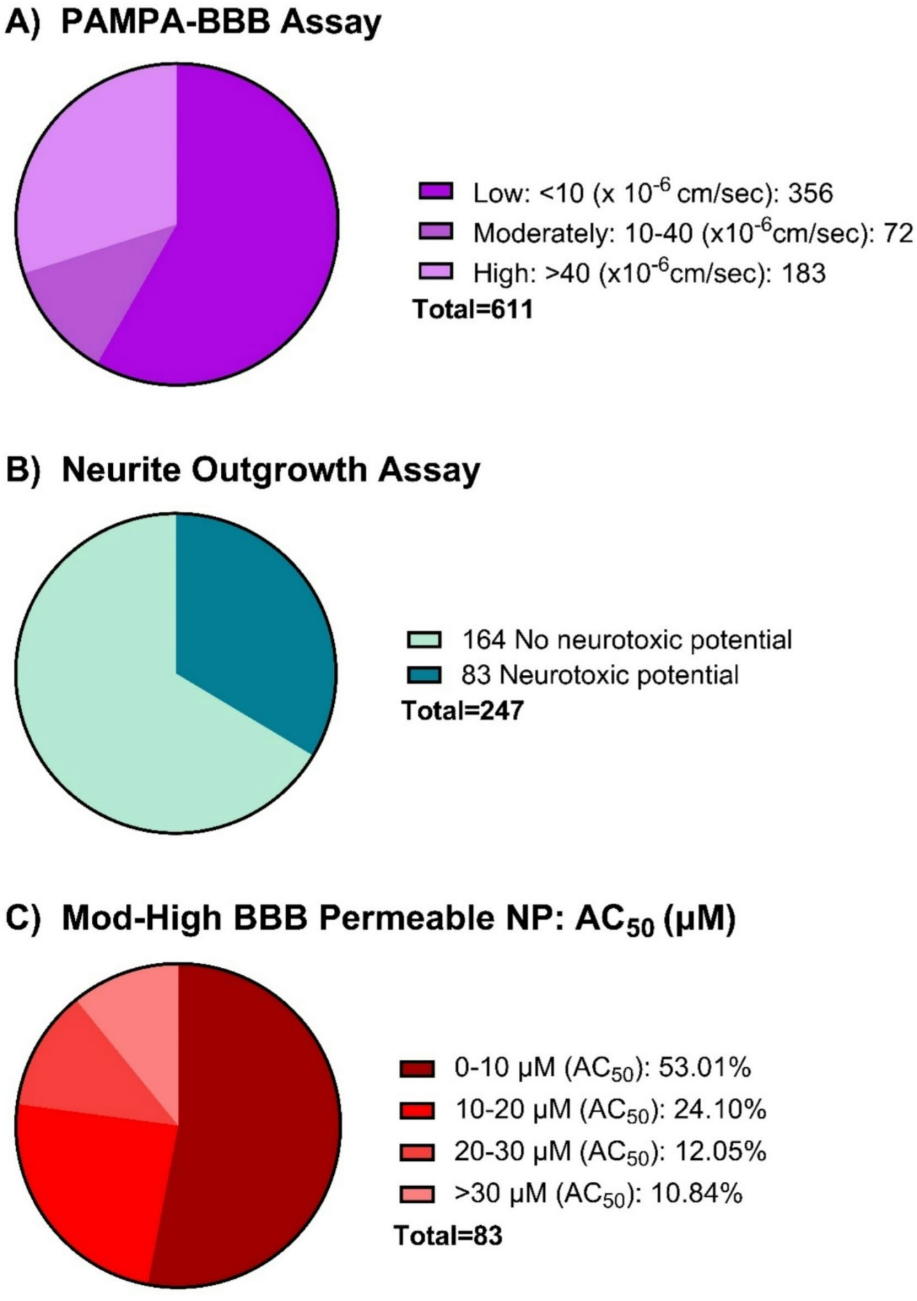
Distribution of the natural product library dataset (**A**) BBB permeability, (**B**) Neurite Outgrowth Assay, and (**C**) AC_50_ distribution for moderate to high permeable constituents.

PAMPA-BBB Assay was performed for over 40 plates in a 96-well format utilizing three control compounds: caffeine (low permeability), carbamazepine (moderate permeability), and progesterone (high permeability) in each plate. More than 1700 NP constituents were tested. Data was obtained for 611 out of the total compounds tested. Undetermined values could be due to one or more of the following reasons: compound precipitation due to insolubility, weak UV signal, non-specific binding or the compound having high affinity to the lipid bilayer. Out of the 611 compounds, 255 (~ 40% of dataset) compounds fell into the moderate to high permeability category (Fig. 1A). We then measured 247 moderate to high permeable compounds using the neurite outgrowth inhibition assay (8 compounds were excluded due to unavailability) for their neurotoxic potential. Among these NP constituents, 83 exhibited neurite outgrowth inhibition (Fig. 1B). More than half of this subset (53.01% of moderate to high permeable and potentially neurotoxic compounds) fell into the 0–10 µM AC_50_ category, while the rest exhibited AC_50_ values > 10 µM (shown in Fig. 1C).

### Molecular property distribution: comparison between NP constituents and NCATS-synthesized compounds

**Fig. 2:**
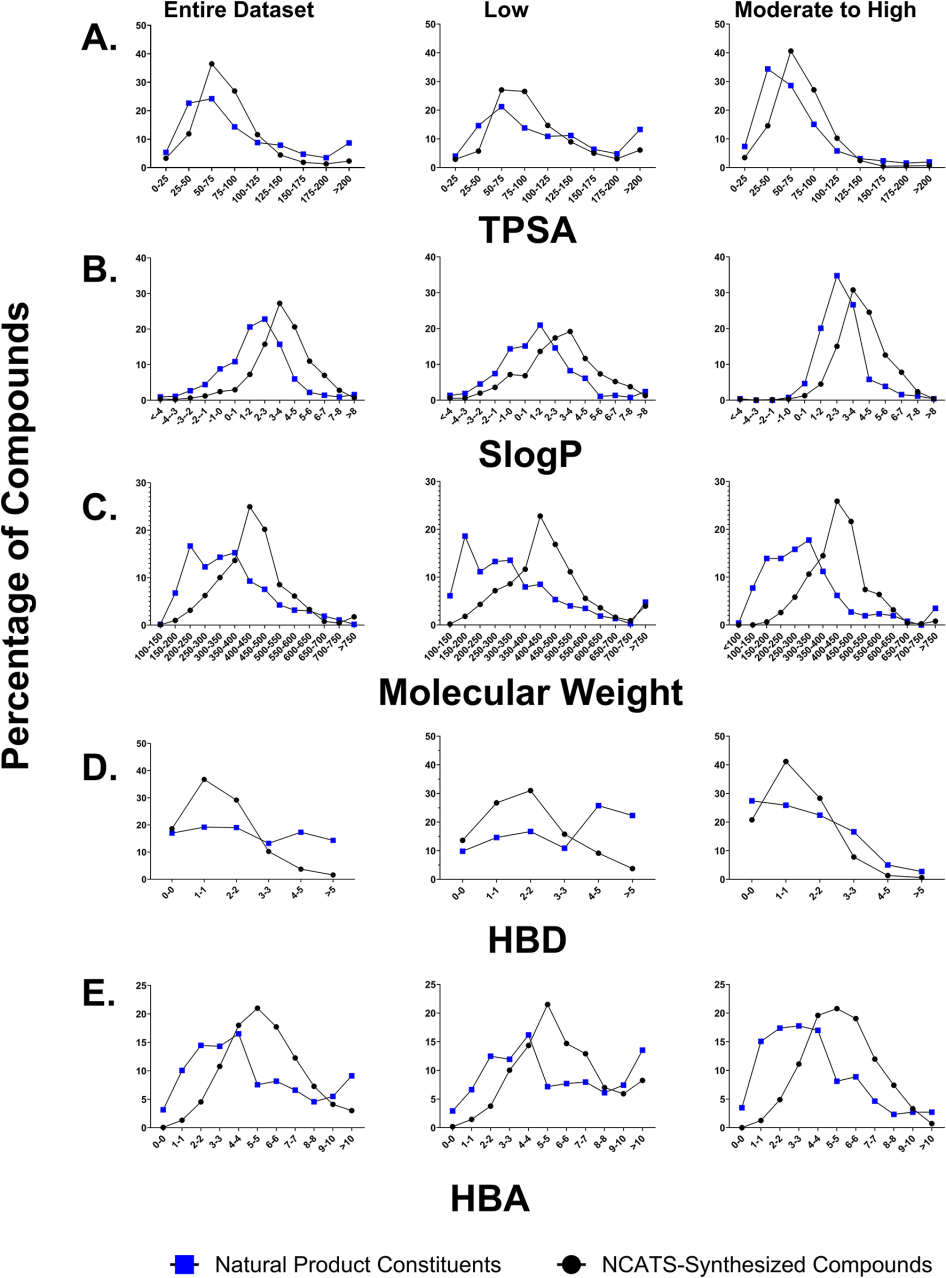
Molecular property distribution plot: Comparing PAMPA-BBB values for NCATS-synthesized compounds for CNS targets versus natural product constituents based on (**A**) TPSA, (**B**) SlogP, (**C**) Molecular Weight, (**D**) Hydrogen Bond Donor, and (**E**) Hydrogen Bond Acceptor.

A physicochemical property correlation between the NCATS-synthesized compounds (~ 2000 compound library synthesized at NCATS for CNS projects^13^) and NP constituents was performed. Molecular properties, including SlogP, total polar surface area (TPSA), molecular weight (MW), hydrogen bond donors (HBD), and hydrogen bond acceptors (HBA), were analyzed to identify any latent trends between NP constituents and NCATS-synthesized compounds. Majority of NP constituents in our dataset belong in the 200–450 molecular weight range, have SlogP values between 0 and 4, TPSA between 25 and 100, and have between 1 and 5 HBA and 0–5 HBD (Fig. 2A-E). Interestingly for both NCATS-synthesized and NP constituent datasets, lower HBD and TPSA compounds tended to have better BBB permeability. Furthermore, both NCATS-synthesized compounds and NP constituents with moderate to high BBB permeability were observed to have higher SlogP values. In contrast, lower HBA values were associated with a higher likelihood of BBB permeation for NP constituents, but this trend was not observed for NCATS-synthesized compounds. Due to the limited size of the natural product library dataset, we avoided drawing definitive conclusions about the observed trends when compared to our NCATS-synthesized compound library. Nonetheless, we conducted additional statistical tests and provided the data in table S4 of the Supplemental Information File 2.

**Fig. 3:**
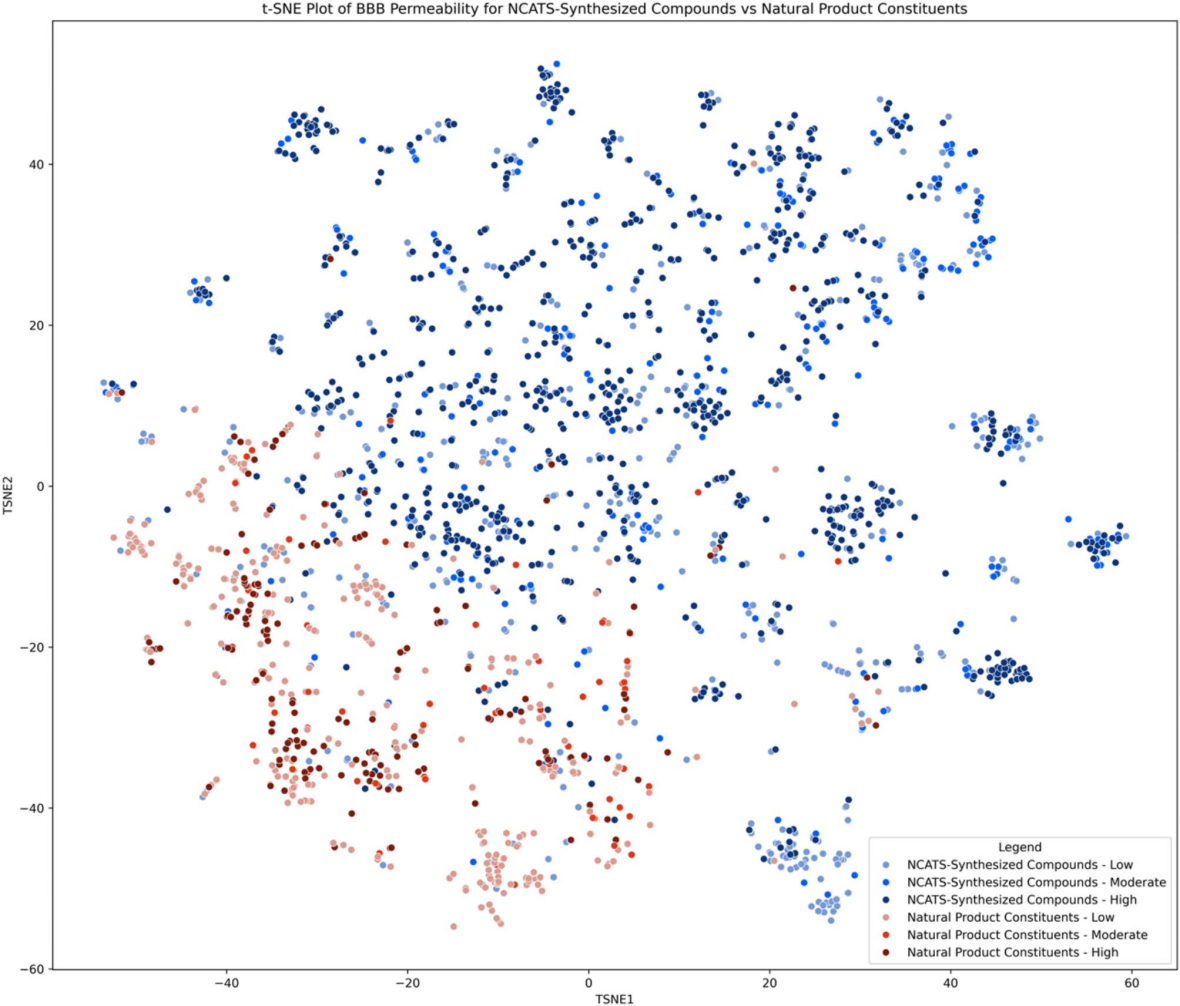
Chemical space visualization of the NP Constituent dataset in comparison to the NCATS-synthesized compound set.

Additionally, visual examination of the chemical space diversity was performed using t-distributed stochastic neighbor embedding (t-SNE) to reduce dimensionality of the molecular properties^25^. Molecular descriptors were calculated from SMILES data via RDKit, with 198 of 210 descriptors retained after removing those that could not be calculated for all molecules. The data was scaled and embedded using Scikit-learn’s StandardScaler and TSNE function respectively, creating a plot where each point represents a molecule (Fig. 3). Colors indicate whether the molecule is NCATS-synthesized (blue) or an NP constituent (red), and color intensity reflects permeability. The t-SNE plot showed distinct grouping of NP constituents and NCATS-synthesized compounds, with some exclusive clusters. However, permeability levels were well distributed across both groups, and it is unclear if adding NP constituents, now ~ 25% of the dataset, would significantly improve model performance (https://opendata.ncats.nih.gov/adme/models/pampa_bbb) due to limited unique chemical space expansion. With the launch of the NIH Helping to End Addiction Long-term (HEAL) initiative (https://heal.nih.gov), NCATS has seen a surge in CNS-targeted projects, for which we regularly rank compounds using the PAMPA-BBB assay. Once our datasets, including NP constituent data, are substantial enough, we plan to re-train the PAMPA-BBB model and deploy it on our in silico website.

### Inhibitory effects of NPs on neurite outgrowth

**Fig. 4:**
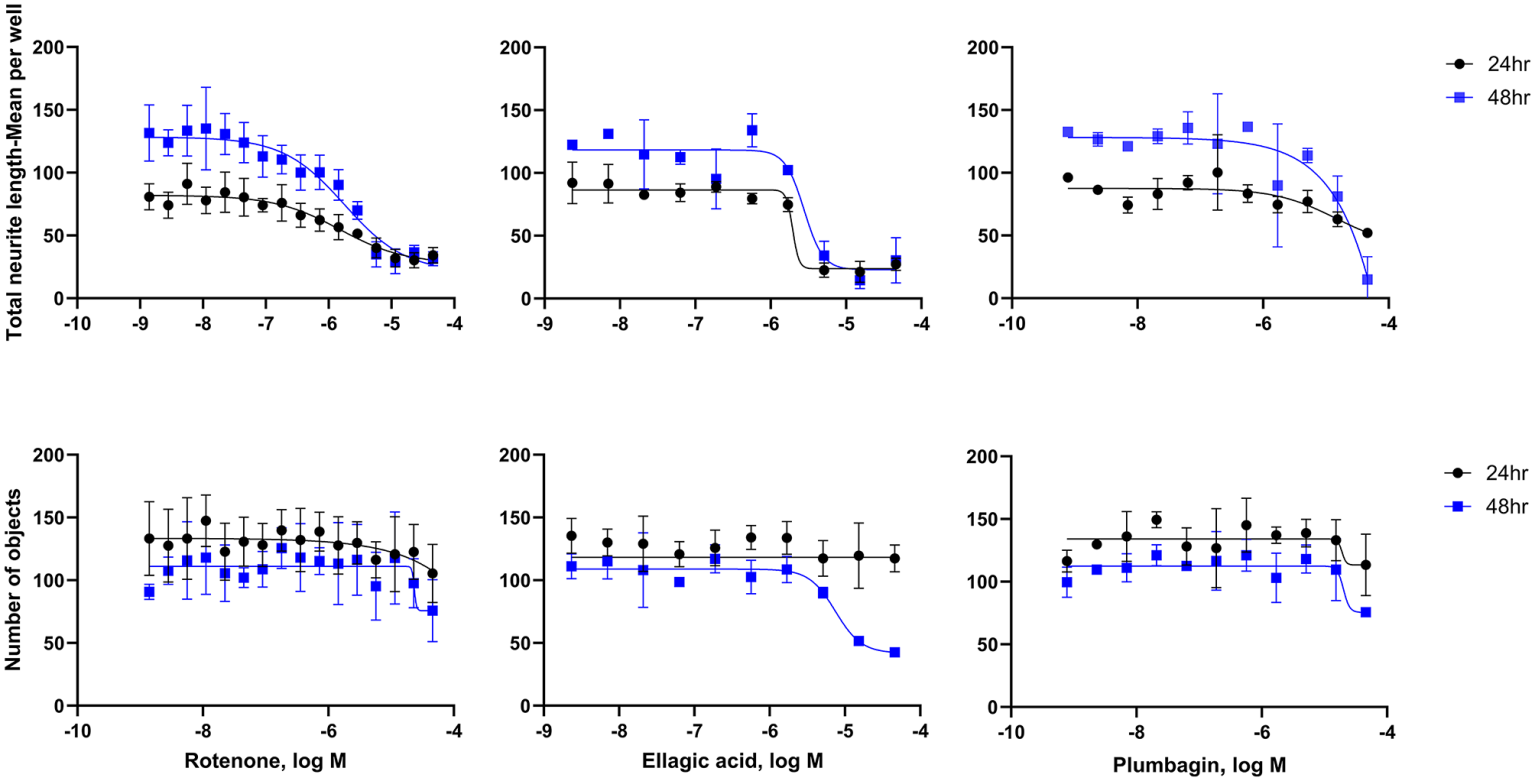
Concentration-response curves for rotenone (positive control), ellagic acid, and plumbagin in cortical neurons. Total neurite length (TNL) and number of objects were measured after 24 (black) and 48 (blue) hour compound treatment.

**Fig. 5:**
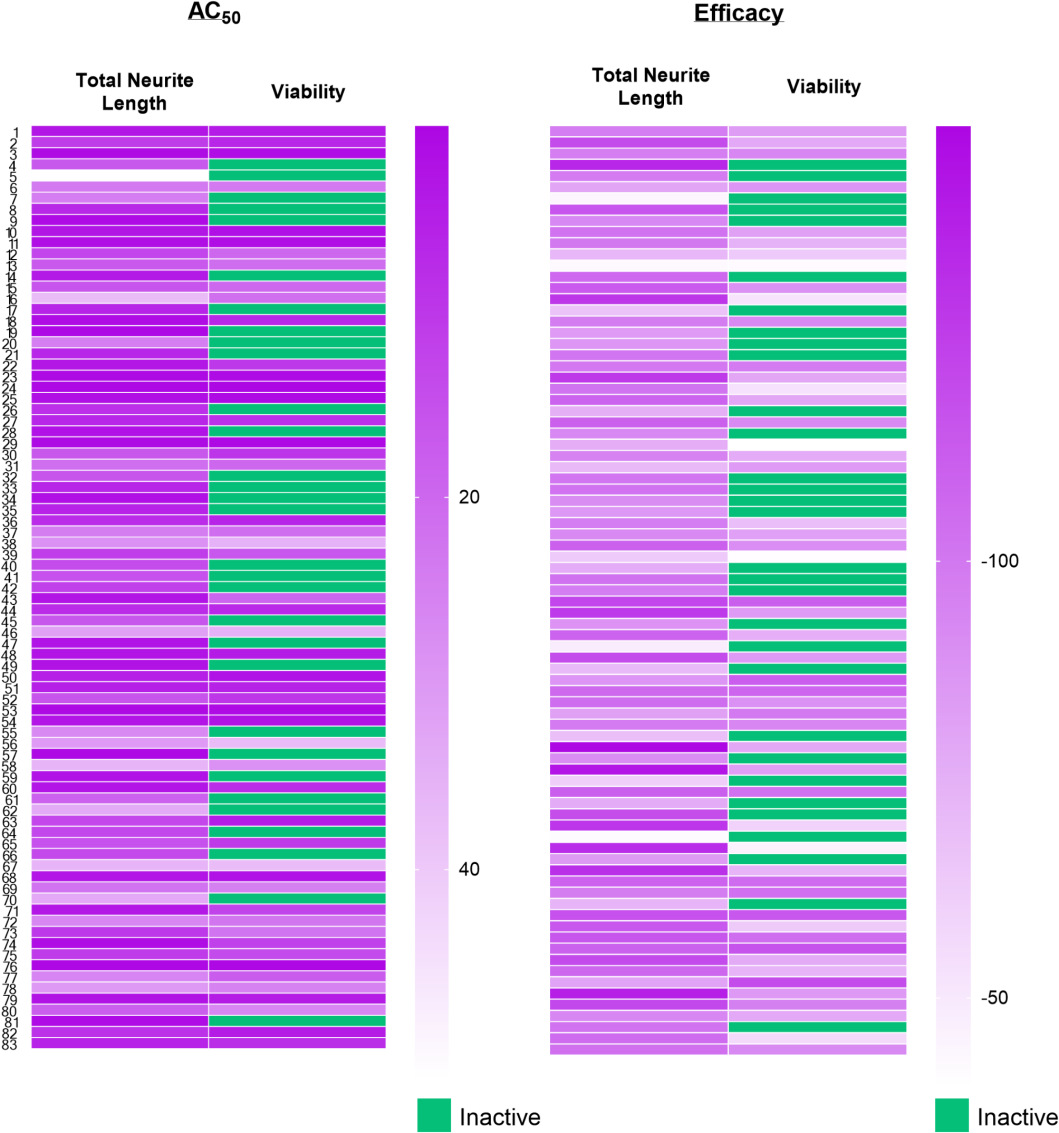
Heat map of all 83 compounds that showed potential for neurotoxicity. GFP labeled cortical neurons were incubated for 24 and 48 h. Endpoints represent total neurite length and viability at 48 h. The compound activity is colored in different shades of purple according to potency (AC_50_ in µM) and efficacy (%). Inactive compounds are colored green.

**Fig. 6:**
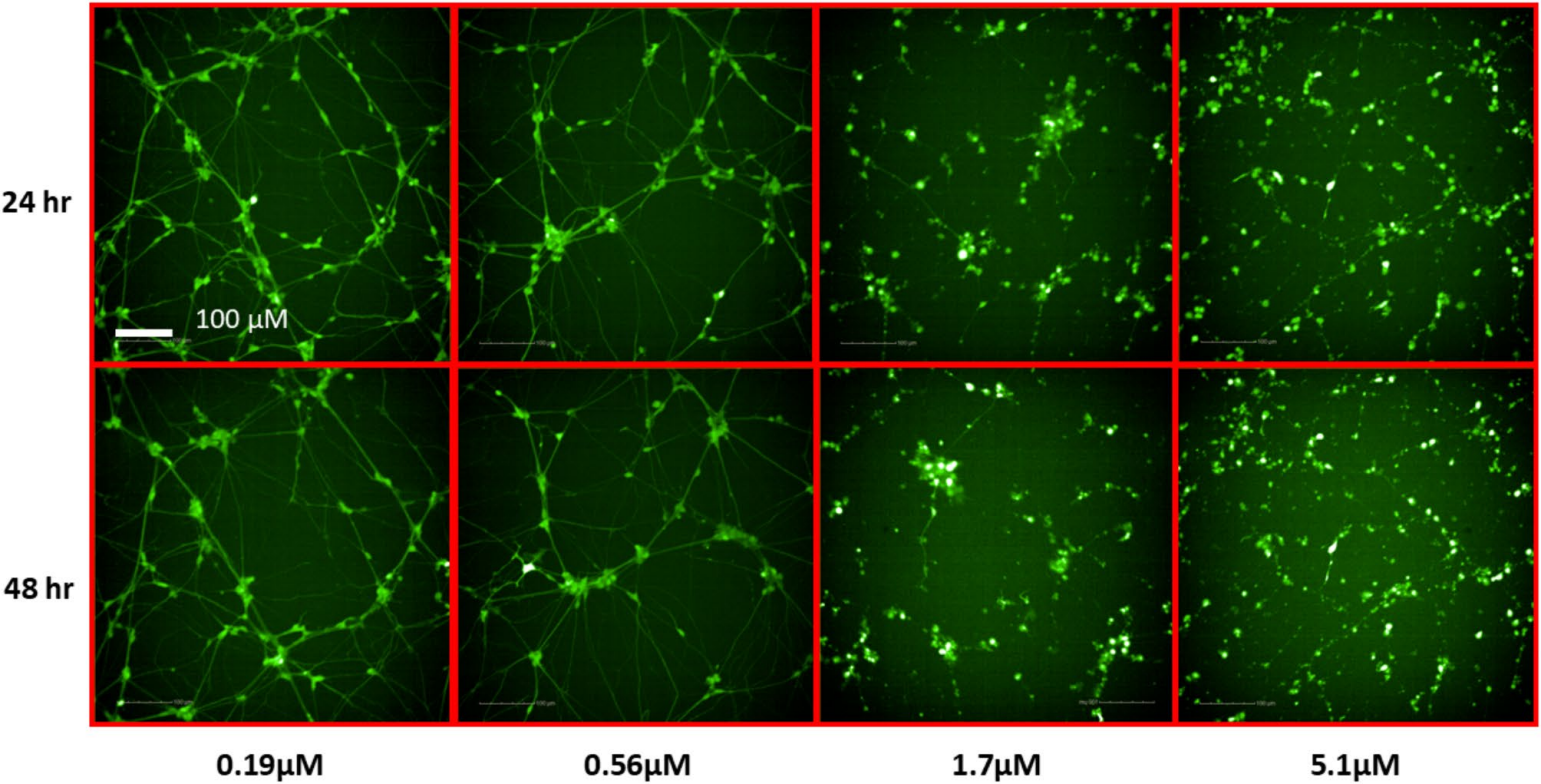
Representative images of cortical neurons after 24 and 48 h treatment with ellagic acid in a neurite outgrowth assay.

**Fig. 7:**
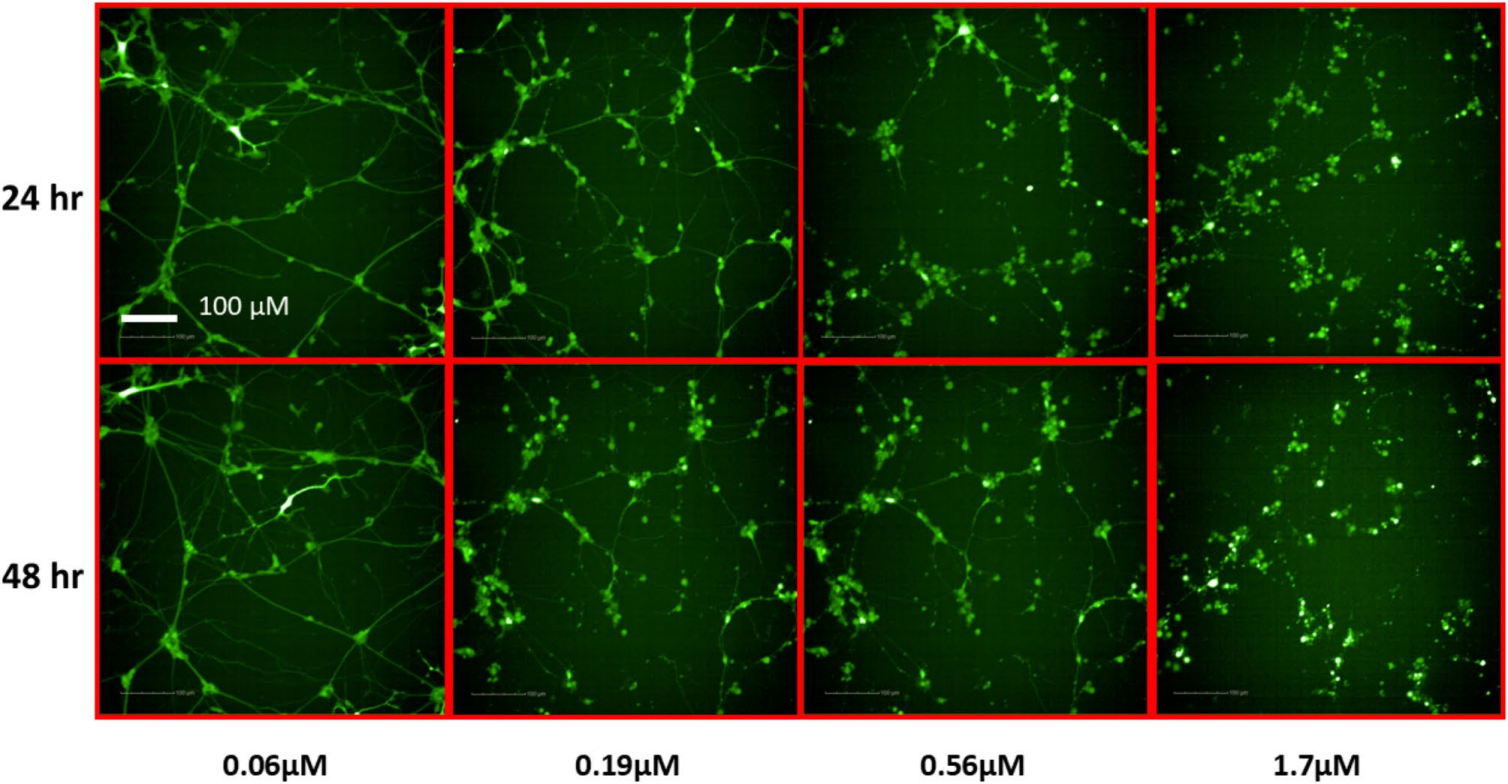
Representative images of cortical neurons after 24 and 48 h treatment with plumbagin in a neurite outgrowth assay.

To identify potentially neurotoxic constituents, a neurite outgrowth inhibition assay was performed. iPSC derived human cortical glutamatergic neurons expressing GFP allow for continual measurement over a 24–48 h period and real time visualization of the neurites in 1536 -well plates^23^. Neurons branch out by growing neurites that contact and link with other surrounding neurons after plating. As a positive control used in this assay, rotenone, a known neurite outgrowth inhibitor, inhibited cortical neurite outgrowth in a concentration-dependent manner (Fig. 4) at both 24 and 48 h time points as reported previously^21^. Eighty-three out of the 247 compounds tested showed inhibitory effects on total neurite length. The top 10 NP constituents that showed neurite outgrowth inhibition, as well as the lowest AC_50_, values include patupilone, combretastatin A4, cinobufagin, cephalomannine, 3’,4’-dimethoxyflavone, plumbagin, noscapine, trichostatin A, chrysoeriol, and sterigmatocystin. (Table 1). Overall, most compounds were observed to have a low AC_50_ value between 0 and 20 µM with ~ 75% efficacy (Fig. 5). However, it was interesting to find that 33 constituents affected only the total neurite length, but not cytotoxicity. On the other hand, 4 constituents show inactivity with respect to total neurite length inhibition but exhibited cytotoxicity. Five out of 10 constituents with the lowest AC_50_ values had no cytotoxic effects. The complete dataset is available in Supplemental Information File 1. High-content images for the represented compounds (i.e. ellagic acid and plumbagin) from a total of 83 toxic compounds are showcased in Figs. 6 and 7. Concentration-response curves of ellagic acid (AC_50_ = 3.1 µM) and plumbagin (AC_50_ = 0.9 µM) are similar to rotenone (Fig. 4), both of these compounds inhibited cortical neurite outgrowth after 24 and 48 h compound treatment, but only had minimal effects on the number of objects after 48 h treatment.

**Table 1:** Summary data for the top 20 potent constituents from the NP constituent datasets.

Name	Origin	PAMPA-BBB Pe (10 × 10^− 6^ cm/sec)	TNL AC_50_ µM (48 h)	Viability AC_50_ µM (48 h)	Target
Patupilone	*Sporangium cellulosum*	10.52	0.007	inactive	Axonal Regeneration^26^
Combretastatin A4	*Combretum caffrum*	315.59	0.028	0.0470	Antimetastatic effects on glioma cells^27^
Cinobufagin	*Bufadienolide*	45.36	0.618	0.093	Inhibit tumor growth in GBM^28^
Cephalomannine	*Taxus yunnanensis*	78.57	0.688	inactive	Anticancer ^29^
3’,4’-dimethoxyflavone	*Primula veris* and *Lawsonia inermis*	249.21	0.759	inactive	Neuroprotective inhibitors of parthanatos^30^
Plumbagin	*Plumbaginaceae* and *Droseraceae*	68.8	0.927	1.321	CNS related (Parkinson’s Disease)^31^
Noscapine	*Papaver somniferum*	74.54	1.064	11.604	Microtubule-inhibitor, potential GBM treatment^32^
Trichostatin A	*Streptomyces*	54.74	1.655	0.472	Neuroprotective effects (Alzheimer’s Disease) by regulating amyloid β plaques^33^
Chrysoeriol	*Schouwia thebaica*, *Perilla frutescens*	178.56	2.221	inactive	CNS related (Epilepsy)^34^
Sterigmatocystin	*Aspergillus nidulans* and *A. versicolor*	221	2.319	inactive	Disruption of Neurogenesis^35^
Ellipticine	*Oschrosia elliptica*,	> 1000	2.644	19.589	Anticancer ^36^
Tacrolimus	Fermented *Streptomyces tsukubaensis*	154.13	2.646	4.190	Neuroprotection^37^
Chelerythrine chloride	Root of *Zanthoxylum simulans*, and *Chelidonium majus L.*	94.67	2.789	2.188	Intracranial tumor suppression (GBM)^38^
NU-7441	Enzyme	101.9	2.849	7.603	Reduced GSC tumor sphere formation (brain)^39^
3-Hydroxyflavone	Papaya, raspberries, black tea, and pomegranate	181.17	2.97	1.835	Neuroprotective Roles (central structures of flavanols)^40^
Ellagic acid	Strawberries, walnuts, cherries, and oak	119.5	3.053	4.002	CNS related (Alzheimer’s Disease)^41^
Harmane	*Passiflora incarnata* (purple passionflower)	59.65	3.212	12.233	Potential psychiatric therapy^42^
Piperlongumine	*Piper longum* (Long pepper fruit)	90.26	3.739	Inactive	Protects aluminum neurotoxicity in zebrafish^43^
Apicidin	*Fusarium pallidoroseum*	55.75	4.838	2.168	Reversing memory deficits (Alzheimer’s Disease)^44^
Celastrol	*Tripterygium wilfordi*	142.58	4.920	5.469	Neuroprotective effects through protecting the BBB^45^

### Summary of CNS-related literature for top 10 potentially neurotoxic compounds

From our study, we not only identified several NP constituents that have the potential to cross the BBB, but we also identified some as having the potential to cause neurotoxicity (i.e. caffeic acid, nootkatone, chrysoeriol, trichostatin A, plumbagin, ellagic acid, apicidin, cinnamyl alcohol, tacrolimus, celastrol, xanthenone, and phenoxodiol). These compounds have been investigated in several different CNS indications, including neuroprotection (Table 1), and it would be interesting to see whether our in vitro neurite inhibition assay results could be replicated in vivo. Here in, we summarize CNS-related literature from our top 10 potentially neurotoxic compounds with additional compound information available in the Supplemental Information File 1.

#### Patupilone AC_50_ (µM) – 0.007

Patupilone is a microtubule-stabilizing drug that comes from the myxobacterium *Sporangium cellulosum*. One study looked at brain metastases of patients with non-small cell lung cancer, and while there has been previous data on BBB permeability, there was no evidence to show a positive effect on the metastases. Patients in both studies discontinued the medication before the trial could be completed because it was poorly tolerated in the body^46^. However, promising results were seen for axonal regeneration after nerve injury. In addition to an increase in motor neuron growth, the study also found some neuroprotective properties, at lower concentrations^26^.

#### Combrestatin-A4 AC_50_ (µM) – 0.028

Combrestatin A4 (CA-4) is a stilbenoid natural product with antimitotic properties derived from the bark of the South African tree *Combretum caffrum*^47^. Studies surrounding glioblastoma multiforme are being investigated, with CA-4 being one of the new agents being considered. CA-4 has exerted potent antiproliferative and apoptotic effects on glioma cells as well as reduced the migration and invasion capabilities of U-87 cells^27^. In another study, CA-4 was utilized to predict BBB delivery of vascular-disrupting agents (VDA) on a metastasized brain. Here, CA-4 was seen to pass the blood brain barrier^48^.

#### Cinobufagin AC_50_ (µM) – 0.6

Cinobufagin is a water decocting extract from *Bufobufo gargarizans cantor*, also known as the Asiatic toad. In China, cinobufagin is still utilized in traditional Chinese medicine Chansu^49^. Cinobufagin has also been studied as a selective anticancer agent against tumors. Specifically, in this study, nude mice with glioblastoma multiforme (GBM), a malignant brain tumor formation, were treated intraperitoneally with cinobufagin. These mice were then subjected to luciferase bioluminescence imaging for 10 days resulting in a 70% decrease in luminescence intensity of the brain tumor. This study implied the monomer, or its metabolites, could cross the BBB therefore inhibiting tumor growth^28^. In another study, cinobufagin’s underlying mechanism to induce cytotoxicity was examined in brain cell models. Results showed that Ca^2+^ rises, leading to Ca^2+^ entry in GHA cells but not in HCN-2 (human cortical neuron) cells due to glial fibrillary acidic protein (GFPA) expression levels^50^.

#### Cephalomannine AC_50_ (µM) − 0.7

Cephalomannine is an alkaloid and a natural congener of paclitaxel^51^. This compound is also known for its potential anticancer properties^52^. Specifically, in one study, cephalomannine was utilized to understand its potential role in lung cancer CNS metastases. Cephalomannine exerts inhibitory effects in hypoxic lung cancer cells, which may be specifically associated with a potential target in the CNS^53^. Cephalomannine was also observed to be cytotoxic in human glial and neuroblastoma cell lines. This study observed taxol, cephalomannine, and 10-deacetylbaccatin III and exposed them in malignant glioma tumor cells. Results showed that all drugs are cytotoxic, however cephalomannine was ~ 35% less cytotoxic than taxol^29^.

#### 3,4-dimethoxyflavone AC_50_ (µM) – 0.7

3,4-dimethoxyflavone (3,4-DMF) is an AhR (a transcription factor responsible for inducing drug-metabolizing enzymes) antagonist found in the leaves of cowslip, *Primula veris* (Pubchem CID: 688674). While commonly used as an anticancer drug, 3,4-DMF was also used to promote proliferation of human hematopoietic stem cells. This research showed promise for using 3,4-DMF in transplantation and gene therapy^54^. Another study looked at the neuroprotective properties of 3,4-DMF by inhibiting parthanatos, a pathway that over-activates PARP-1, leading to neuronal death^30^.

#### Plumbagin AC_50_ (µM) – 0.9

Plumbagin is a naphthoquinone obtained from the roots of different medicinal plant families, such as Plumbaginaceae, Droseraceae, and Ebenaceae. *Plumbago zeylanica L*. has been considered the most known medicinal plant that contains plumbagin^55^. Plumbagin has shown counteracting effects in human inflammation models and other pharmacological properties including antitumor, antioxidation, and neuroprotective effects. Plumbagin has been investigated for its potential against Parkinson’s disease both in vitro and in vivo, demonstrating inhibition of the TLR/NF-κB pathways^31^. Additionally, studies have shown that plumbagin inhibits neuronal apoptosis, reduces intimal hyperplasia, and suppresses the TNF-α/NF-κB pathway, which is implicated in CNS injury^56^.

#### Noscapine AC_50_ (µM) – 1.1

Noscapine is a phthalideisoquinoline that is obtained from opium harvesting^57^. Studies have shown that noscapine can effectively cross the BBB. One study found neuroprotective properties, as well as little evidence of toxicity on dorsal root ganglia; however, vasodilation was noticed in treated brain tissue^58^. Noscapine also showed neuroprotective properties in another study, which tested its effects on Parkinson’s disease. As oxidative stress and neuroinflammation play vital roles in Parkinson’s disease, promising results were seen in decreasing the upregulation of both pro-inflammatory factors and oxidative stress, as well as inhibiting apoptosis^59^.

#### Trichostatin A AC_50_ (µM) – 1.7

Trichostatin A (TSA) is a hydroxamic acid initially isolated from the metabolites of *Streptomyces hygroscopicus* strains. TSA shows various pharmacological properties such as antioxidant, antidiabetic, anti-inflammatory, and anticancer^60^. TSA is a histone deacetylase inhibitor, and in addition to these beneficial properties, it also exhibits neuroprotective effects. The compound alleviated the spatial learning and memory deficits in mice with early-onset Alzheimer’s symptoms^33^. Another study on the brain showed TSA relieving meningitis and ameliorating cognitive impairments in infected mice^61^.

#### Chrysoeriol AC_50_ (µM) – 2.2

Chrysoeriol is a flavone which is found in several plant species. The structure of chrysoeriol is 30-O-methoxy flavone, a derived product of luteolin. Some of the plant species it stems from include *Caoronopus didymus*,* Capsicum sp.*,* and Eurya cilliata*^62^. Chrysoeriol has been shown to activate Nrf2 in other cell types, and has been reported to have anti-inflammatory activity^63^. In another study, chrysoeriol was reported to reduce reactive oxygen species accumulation and upregulate mitochondrial-related transcript expressions such as PGC1-Alpha^64^, which has shown protective effects via the mitochondrial pathway in rat brains after intracerebral hemorrhages^65^.

#### Sterigmatocystin AC_50_ (µM) – 2.3

Sterigmatocystin (STC) is a natural product found in *Aspergillus amstelodami*, *Aspergillus multicolor*, and other organisms^66^. STC has been identified in grains, coffee beans, nuts, beer, spices, and cheeses^67^. Studies have shown concerns with developmental neurological disorders when exposed to STC. However, no adverse effects on offspring neurogenesis were observed from maternal oral exposure to STC at 5.0 ppm^35^. In another study, STC was evaluated for human neurotoxicity. As a mycotoxin, STC was observed to harm neuronal cells via oxidative stress as well as induce cytotoxicity^68^.

## Discussion

CNS drug discovery and development is a particularly challenging endeavor, as evidenced by the lower probability of these drugs reaching the market compared to the pharmaceutical industry average across other target areas^69,70^. This is also reflected in the fact that it takes longer to bring a CNS drug into the market (12–16 years) compared to a non-CNS drug (10–12 years)^71^. Lack of BBB penetration is one of the top reasons for these low success rates. The clinical development of tarenflurbil (treatment for Alzheimer’s), was recently halted after it failed to show efficacy in a Phase III clinical trial. A multicenter, randomized, double-blind, placebo-controlled study demonstrated that after 18 months of treatment, tarenflurbil did not slow cognitive decline. Poor BBB-penetration and inadequate target engagement in the brain were identified as the causes of failure^72^. Another example is Gavestinel, which reached Phase III clinical trials but did not show clear effectiveness, likely due to its inability to cross the BBB^71,73^.

Failures in drug development are always disheartening, but the most challenging situation arises when these failures are identified at an advanced stage after many years of research and significant financial investment. Therefore, it is essential to address the question of whether a drug can penetrate the BBB early in the drug discovery process. The emergence of high-throughput screening and the resulting surge in the number of potential hits have made it impractical to test all promising compounds in vivo using pre-clinical models. Various cell-based and non-cell-based in vitro assays, such as MDCK-MDR1 cells, Caco-2 cells, PAMPA-BBB, and Organ-on-a-chip assays, are frequently used to rank and prioritize compounds for further progression to animal studies^13^. PAMPA-BBB is routinely used for rank-ordering compounds in drug discovery because of its robust, adaptable, and high-throughput nature, with our previous study showing an approximate 77% categorical correlation between in vitro PAMPA-BBB and in vivo data in rodents.

Drugs can reach the brain through various mechanisms. Passive diffusion allows small, lipophilic molecules to cross the BBB, while active and carrier-mediated transport rely on specialized proteins to move substances across. Larger molecules may use receptor-mediated transcytosis, and positively charged drugs can leverage adsorptive-mediated transcytosis. Advanced techniques like nanoparticle delivery and direct administration bypass the BBB, and temporary BBB disruption can facilitate drug entry for larger or less permeable compounds^74^. One of the major caveats of the PAMPA-BBB assay is that it can only model passive transport. This disadvantage is offset by the fact that majority of compounds that penetrate the BBB do so passively^75,76^. It is essential to recognize that compounds demonstrating high permeability in our PAMPA-BBB assays may still encounter efflux issues, potentially preventing them from crossing the BBB in vivo. Likewise, compounds that exhibit low BBB permeability in our PAMPA-BBB assays may still cross the BBB via alternative mechanisms. Thus, it is essential to assess a compound’s potential to cross the BBB through multiple complementary assays, while continually comparing in vitro results with in vivo brain penetration data to keep project teams aligned and informed.

Recently, compelling evidence has highlighted the significant role of gut microbiota in the bidirectional interactions between the gut and the nervous system. The gut-brain axis is a bi-directional communication network linking the gastrointestinal (GI) tract and the CNS through neural, hormonal, immune, and biochemical signals. The gut microbiota, comprising trillions of microorganisms, significantly impacts this interaction by producing metabolites that influence brain function, while the brain can affect gut activity and microbiota composition. Metabolites produced in the colon through bacterial fermentation of dietary fibers and resistant starch, such as short-chain fatty acids (SCFAs) like acetate, propionate, and butyrate, have been shown to exert neuromodulatory effects^77–79^. An intriguing example is the case of glucagon-like peptide-1 (GLP-1) agonists. GLP-1 is primarily produced and secreted by enteroendocrine cells in the intestines, particularly in the ileum and colon, in response to food intake. Initially developed for treating type 2 diabetes, it was soon recognized that GLP-1 agonists provide several additional physiological benefits. These include neuroprotective, neurotrophic, and anti-inflammatory effects, which could play a role in slowing the progression of Alzheimer’s disease^80^. This axis is crucial for homeostasis and is linked to mental health, neurodegenerative diseases, and digestive disorders^81–83^. Drug discovery in this area aims to modulate the gut-brain connection, opening pathways for new treatments for complex conditions involving both systems. This makes CNS drug discovery even more complex since drugs can potentially target the CNS through the gut-brain axis without necessitating BBB penetration.

Neurite outgrowth assays are valuable in neurobiology research and in drug discovery for identifying compounds that promote neural growth or protect against neurodegenerative conditions. However, their relevance to in vivo conditions in animals is limited by the complexity of living systems, which involve factors like immune responses, systemic interactions, and multi-cellular environments not fully captured in vitro. While these assays provide essential initial insights, further validation in animal models is needed to confirm their effects in a more complex, integrated system^84^.

NPs have remained a promising cache of compounds for therapeutic potential in CNS drug discovery with their vast diversity and structural complexity^85^. However, the lack of BBB penetration data is one of the key hurdles that continues to challenge the drug discovery community. Therefore, understanding BBB penetration is critical to streamline and optimize CNS drug discovery. Conversely, it is crucial to assess the potential for neurotoxicity at an early stage, especially given their widespread and unregulated use^86,87^. In this regard, we anticipate that our datasets will significantly impact researchers working with NP constituents. Although this data is highly intriguing, it will be very valuable to further investigate these constituents in vivo to understand their disposition and toxicity. Additionally, the collected data will be incorporated into the next iteration of our in silico PAMPA-BBB model to enhance the dataset’s diversity (https://opendata.ncats.nih.gov/adme/) and support the broader drug discovery community.

## Electronic supplementary material

Below is the link to the electronic supplementary material.

Supplementary Material 1

Supplementary Material 2

## Data Availability

All data has been made available in the Supplemental Information File 1.
