# Supplementary material for: Investigating blood–brain barrier penetration and neurotoxicity of natural products for central nervous system drug development
Source: Sci Rep. 2025 Mar 3;15:7431. doi: 10.1038/s41598-025-90888-2 (PMC11876671; doi:10.1038/s41598-025-90888-2)
Supplement: Supplementary file 2 — Supplementary Material 2 [file 41598_2025_90888_MOESM2_ESM.pdf]

Supplemental Information

Investigating Blood-Brain Barrier Penetration and Neurotoxicity of Natural Products for Central Nervous System Drug Development

Rintaro Kato, Li Zhang, Nivedita Kinatukara, Ruili Huang, Abhinav Asthana, Claire Weber, Menghang Xia, Xin Xu, and Pranav Shah*

National Center for Advancing Translational Sciences (NCATS), 9800 Medical Center Drive, Rockville, Maryland 20850, United States

*Corresponding Author: Pranav Shah, pranav.shah@nih.gov

Table S4: Comparison of molecular descriptors between different PAMPA-BBB categories for NP constituents and NCATS-synthesized compounds. Data shown as mean ± SD. A two-tailed t-test with unequal variance was performed to determine statistical significance

	**TPSA**	**SLogP**	**HBD**	**HBA**	**MW**
**NP Low**	115.15 ± 89.14	1.31 ± 2.64	3.86 ± 3.44	5.89 ± 4.57	350.47 ± 214.51
**NP Mod-High**	68.27 ± 54.52	2.81 ± 1.57	1.66 ± 2.08	3.82 ± 2.95	320.91 ± 172.79
**p-Value**	<0.001	<0.001	<0.001	<0.001	0.055
**NP Low**	115.15 ± 89.14	1.31 ± 2.64	3.86 ± 3.44	5.89 ± 4.57	350.47 ± 214.51
**NCATS Low**	102.11 ± 67.85	2.82 ± 2.59	2.13 ± 2.31	6.15 ± 3.53	454.38 ± 184.69
**p-Value**	0.016	<0.001	<0.001	0.34	<0.001
**NP Mod-High**	68.27 ± 54.52	2.81 ± 1.57	1.66 ± 2.08	3.82 ± 2.95	320.91 ± 172.79
**NCATS Mod-High**	73.80 ± 33.93	4.01 ± 1.56	1.33 ± 1.20	5.26 ± 2.04	432.09 ± 107.46
**p-VALUE**	0.117	<0.001	0.014	<0.001	<0.001
**NCATS Low**	102.11 ± 67.85	2.82 ± 2.59	2.13 ± 2.31	6.15 ± 3.53	454.38 ± 184.69
**NCATS Mod-High**	73.80 ± 33.93	4.01 ± 1.56	1.33 ± 1.20	5.26 ± 2.04	432.09 ± 107.46
**p-Value**	<0.001	<0.001	<0.001	<0.001	0.008
